# Genomes2Drugs: Identifies Target Proteins and Lead Drugs from Proteome Data

**DOI:** 10.1371/journal.pone.0006195

**Published:** 2009-07-10

**Authors:** David Toomey, Heinrich C. Hoppe, Marian P. Brennan, Kevin B. Nolan, Anthony J. Chubb

**Affiliations:** 1 Molecular Modelling Group, Royal College of Surgeons in Ireland, Dublin, Ireland; 2 CSIR Biosciences, Pretoria, South Africa; 3 Pharmaceutical and Medicinal Chemistry, Royal College of Surgeons in Ireland (RCSI), Dublin, Ireland; University of Cincinnati, United States of America

## Abstract

**Background:**

Genome sequencing and bioinformatics have provided the full hypothetical proteome of many pathogenic organisms. Advances in microarray and mass spectrometry have also yielded large output datasets of possible target proteins/genes. However, the challenge remains to identify new targets for drug discovery from this wealth of information. Further analysis includes bioinformatics and/or molecular biology tools to validate the findings. This is time consuming and expensive, and could fail to yield novel drugs if protein purification and crystallography is impossible. To pre-empt this, a researcher may want to rapidly filter the output datasets for proteins that show good homology to proteins that have already been structurally characterised or proteins that are already targets for known drugs. Critically, those researchers developing novel antibiotics need to select out the proteins that show close homology to any human proteins, as future inhibitors are likely to cross-react with the host protein, causing off-target toxicity effects later in clinical trials.

**Methodology/Principal Findings:**

To solve many of these issues, we have developed a free online resource called Genomes2Drugs which ranks sequences to identify proteins that are (i) homologous to previously crystallized proteins or (ii) targets of known drugs, but are (iii) not homologous to human proteins. When tested using the *Plasmodium falciparum* malarial genome the program correctly enriched the ranked list of proteins with known drug target proteins.

**Conclusions/Significance:**

Genomes2Drugs rapidly identifies proteins that are likely to succeed in drug discovery pipelines. This free online resource helps in the identification of potential drug targets. Importantly, the program further highlights proteins that are likely to be inhibited by FDA-approved drugs. These drugs can then be rapidly moved into Phase IV clinical studies under ‘change-of-application’ patents.

## Introduction

The modern molecular biologist is confronted with increasingly large datasets. Genome sequencing data, proteomics data and microarray data are increasingly accessible, but difficult and laborious to interpret. Considering the investment cost of target validation, one needs to rank genome-sized output data in favour of proteins that can readily be modelled using homology modelling, as these structural models can be used in virtual high throughput screening (vHTS) of large compound libraries [1]–[3]. Microbiologists designing antibiotics need to rank their candidate proteins for lack of similarity with any human protein, to reduce the possibility of potentially toxic off-target side effects due to cross-reactivity between inhibitors and patient host proteins. In addition, it is now possible to screen the proteome for homology to targets of known drugs, using the DrugBank dataset [4], and propose FDA-approved drugs for rapid development to Phase IV clinical trials as these compounds are all defined as safe for human consumption. Much of the necessary search functionality is already available online [4]–[7]. However, the assimilation of this data into a cohesive table for analysis is non-trivial for molecular biologists unskilled in programming languages or database management. By providing a convenient online interface and summary table output, we hope to make this analysis open to a wide research audience.

## Materials and Methods

Genomes2Drugs was developed using open source Java Enterprise Edition in the NetBeans IDE 6.0 programming environment and deployed on Sun Application Server [8]. The Basic Local Alignment Search Tool (BLAST) program 2.2 was obtained from the USA National Center for Biotechnology Information (NCBI). The human genome protein sequences and PDB protein sequences were also obtained from NCBI. Drug target protein sequences were obtained from the University of Alberta DrugBank website [4]. Output data files are parsed using BioJava 1.6 and the data entered into an open source MySQL 5.1 database. The test genome *Plasmodium falciparum* 3D7 protein sequences were obtained from the European Molecular Biology Laboratory - European Bioinformatics Institute (EMBL-EBI) Integr8 website (493.P_falciparum, [9]).

## Results

**Figure 1:**
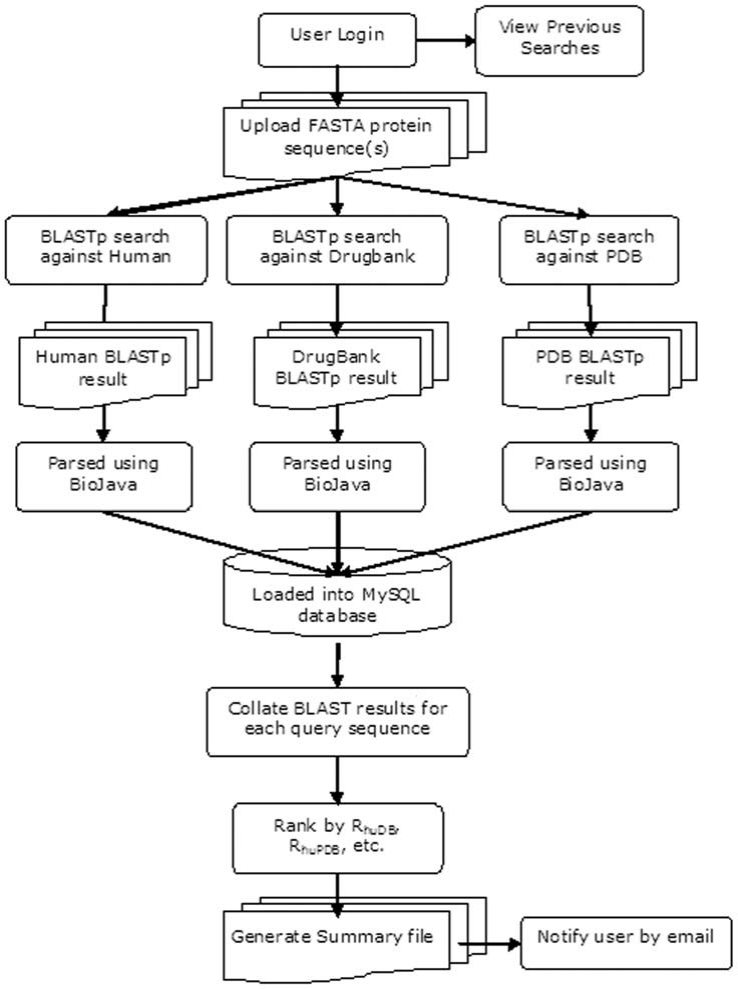
Schema of data processing. Genomes2Drugs is a free online resource. The web interface was written using open-source Java Enterprise Edition, BioJava 1.6 and NetBeans IDE 6.0. Input sequences are aligned against the human proteome, the PDB dataset and the DrugBank target proteins dataset. Only the best results are preserved. The resulting output files are parsed using BioJava and entered into a MySQL 5.1 database, where the results are sorted and ranked. Output XML files are generated from this data.

Genomes2Drugs is a freely available web-based search engine that simultaneously searches each input protein sequence against the protein sequences of the human genome, the DrugBank dataset drug targets and the PDB protein structure database [http://mmg.rcsi.ie:8080/g2d/]. The schema for information processing is shown in Figure 1. Users can input either a single FASTA formatted protein sequence [10] or multiple sequences, either in an input box or an uploaded text file. For instance, complete proteome sequences can be downloaded from the EMBL-EBI Integr8 website [9], and uploaded into Genomes2Drugs. Screen shots of the input and output screens are shown in supplementary Figure S1 online. Users need to register and submit an email address, as processing occurs in the background. User information will remain private and will not be given to any third party. The user will be emailed when the job is complete, and can then login to download the result XML file which can be imported into Microsoft Excel as a ‘As an XML list’, provided the user has downloaded the ‘g2d.xsd’ file (available online) into the same directory. The results from a few input polypeptides can be opened in Excel, while larger genome wide searches should be opened in a database viewer like Microsoft Access, for which a viewing form is included (see supplementary Figure S1B). For easy of access to the data in Access we have included a template MDB file and XSD schema file which need to be downloaded to the same directory as the XML file. The output terms are described in Table 1. Each E_BLASTp_ value is derived from the optimal alignment across the genome using default settings of NCBI's freely available BLASTp algorithm [5], [6]. As the best alignment score is recorded for each input protein, it follows that a poor score indicates that there is no matching protein in the comparator set. Thus a large E_BLASTp_[query vs human genome] value indicates that there is likely no match for that query protein in the human genome. Similarly, good sequence identity, with a small E_BLASTp_[query vs PDB] value indicates that the query sequence has a close homologue in the PDB structural database. No lower limit is set for any E value during the alignment calculation and only the best results are shown.

**Table 1:** Key for output file column headings.

Column title	Explanation
query_id	Unique query entry number.
query_accession	First word of input protein title.
query_title	Input protein title after ‘〉’.
query_length	Number of residues in input sequence.
RhuDB	Logarithm (base 10) of the ratio of 〈human expect〉 and 〈drugbank expect〉.
RhuDBRank	Entries ranked by descending R_huDB_.
RhuPDB	Logarithm (base 10) of the ratio of 〈human expect〉 and 〈PDB expect〉.
RhuPDBRank	Entries ranked by descending R_huPDB_.
RDBPDB	Logarithm (base 10) of the ratio of 〈drugbank expect〉 and 〈PDB expect〉.
RDBPDBRank	Entries ranked by descending R_DBPDB_.
human_accession	First word of human protein title.
human_title	Extracted from target sequence name in BLASTp output.
human_expect	Only optimal human/query alignment is returned, i.e. lowest BLASTp E value.
human_rank	Query vs Human genome alignments are ranked by descending 〈human_expect〉. I.e. poor/no match to the human genome is scored well and given a low rank number.
human_identities	Number of identical residues in query and human sequences.
human_percent_identities	(〈human identities〉/〈query length〉)*100.
human_positives	Number of homologous residues in query and human sequences.
human_percent_positives	(〈human positives〉/〈query length〉)*100.
pdb_accession	Protein Data Bank accession number: pdb¦xxxx¦x
pdb_title	Name of protein 3-D structure.
pdb_expect	Only optimal PDB/query alignment is returned, i.e. lowest BLASTp E value.
pdb_rank	Query vs Protein Data Bank sequence alignments are ranked by ascending 〈pdb_expect〉. I.e. excellent matches with very low E values are scored well and given a low rank number.
pdb_identities	Number of identical residues in query and PDB sequences.
pdb_percent_identities	(〈pdb_identities〉/〈query length〉)*100.
pdb_positives	Number of homologous residues in query and PDB sequences.
pdb_percent_positives	(〈pdb_positives〉/〈query length〉)*100.
drugbank_accession	DrugBank accession number of target protein: nnnn_all_target_protein.fasta.
drugbank_title	Name of DrugBank target protein, including target drug accession numbers in parentheses: (DBnnnnn).
drugbank_expect	Only optimal DrugBank/query alignment is returned, i.e. lowest BLASTp E value.
drugbank_rank	Query vs DrugBank sequence alignments are ranked by ascending 〈pdb_expect〉. I.e. excellent matches with very low E values are scored well and given a low rank number.
drugbank_identities	Number of identical residues in query and DrugBank sequences.
drugbank_percent_identities	(〈drugbank_identities〉/〈query length〉)*100.
drugbank_positives	Number of homologous residues in query and DrugBank sequences.
drugbank_percent_positives	(〈drugbank_positives〉/〈query length〉)*100.

The 〈human expect〉 and 〈PDB expect〉 columns can be used individually to rank the whole input genome for proteins showing little homology to the human genome or good homology to a protein for which the crystal structure has been determined, respectively. More conveniently, the ratio of these expect values can be used to rank the output list according to proteins that would be readily structurally modelled, while also showing little identity to any human proteins. This ratio is provided in the logarithmic (base 10) form, in the column R_huPDB_ (2), which has been ranked by descending value.

The ratio values are calculated as follows:
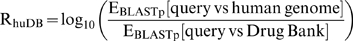
(1)

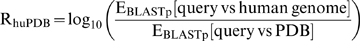
(2)

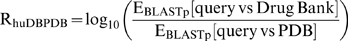
(3)Where E_BLASTp_[] is the expect value extracted from the BLASTp alignment output file using open-source BioJava [8]. The BLASTp algorithm approximates the best alignment (E value  =  1e-180) to zero. To include these data in the ratios, we set E = 0.0 back to E = 1e-180. To include the important ‘NULL’ results from the human search in our ratio calculations, we arbitrarily set this to 1000. The full range for the R_huDB_ and R_huPDB_ values is thus −183 to +183. However, a ‘NULL’ result from the PDB and DrugBank database searches needs to be flagged, as these query proteins are likely to be more difficult to homology model, and do not show homology to targets of known drugs. Error messages from these ratios are defined in Table 2. The negative numbers used will rank these queries to the bottom a descending list.

**Table 2:** Definition of ratio ranges and error codes.

	R_huDB_	R_huPDB_	R_DBPDB_
E_BLASTp_[hum]ψ vs. E_BLASTp_[DB/PDB]ξ	−183 to 183	−183 to 183	−7000
E_BLASTp_[hum]ψ vs. ‘Null’ DB/PDBϕ	−2000	−5000	−8000
‘Null’ DB/PDBϕ vs. E_BLASTp_[hum]ψ	−3000	−6000	−9000

ψ BLASTp expect value of the best query/human genome alignment (null = 1000).

ξ BLASTp expect value of the best query/DrugBank alignment or query/protein data bank alignment (not null).

ϕ No alignment found between query and either DrugBank or PDB databases (null).

Query sequences that show good homology to crystal structure template sequences, but poor/no homology to any protein within the human genome, will have high R_huPDB_ values. The researcher may be particularly interested in the “hypothetical” or “unknown” query proteins that are ranked well according to R_huPDB_ (in the top ∼100) as these may make excellent targets for novel research into characterisation, validation, crystallography/modelling and virtual high throughput screening.

**Figure 2:**
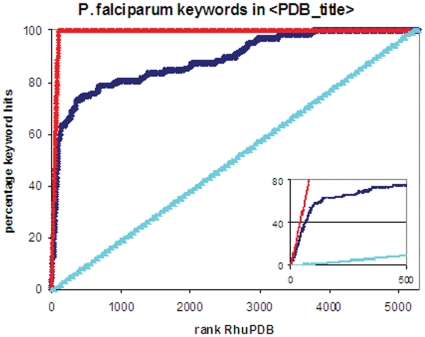
Enrichment of *P. falciparum* proteome by R_huPDB_ – PDB targets. Enrichment curves plot the accumulation of user-defined ‘hits’ as a function of rank number. Thus in an ideal case (red line), each consecutive entry in the ascending ranked list will be a hit. Alternatively, if ranking provides no selection the hits will be distributed randomly across the genome (light blue line). The enrichment percentage as a function of rank are shown in dark blue. The 5283 proteins in the *P. falciparum* 3D7 strain test set were searched using Genomes2Drugs and ranked by R_huPDB_. *P. falciparum* and malaria related hits from PDB were identified using keyword searching of the 〈pdb_title〉 field, and their position in the ranked list identified. The insert, which highlights the first 500 entries, shows that almost 80% of the entries with close homology to known *P. falciparum* crystal structures were identified in the first 10% of the genome.

A sample output from a search using the full proteome of the malaria parasite, *Plasmodium falciparum*, is shown in supplementary Table S1 online. The 5283 FASTA formatted protein sequences in the malarial genome were downloaded from the EMBL-EBI Interg8 website [9] and used as a test set. Of the top 50 entries as ranked by R_huPDB_, the majority (68%) showed previous investigation and/or homology to crystal structures of *Plasmodium falciparum* proteins, indicating that this simple ranking system highlights good candidate drug targets (see Figure 2). This is further illustrated over the full genome test set in Figure 2. A query entry was defined as a ‘hit’ if the PDB title contained keywords associated with malaria. After ranking all 5283 test set entries according to R_huPDB_, the percentage of hits found is plotted as a function of rank number. Thus in the insert in Figure 2 it is clear that ∼80% of the hits are recovered within the first 500 entries, or 10% of the genome. The red line in Figure 2 shows an ideal case where each consecutive entry is a hit, while the light blue line shows a random distribution of hits. Interestingly, 25 of the top 50 entries are uncharacterised “hypothetical”, “putative” or “unknown” proteins, which warrant further investigation as novel drug targets by virtue of the fact that they are (i) pathogen specific and (ii) similar to a structural template for homology modelling.

**Figure 3:**
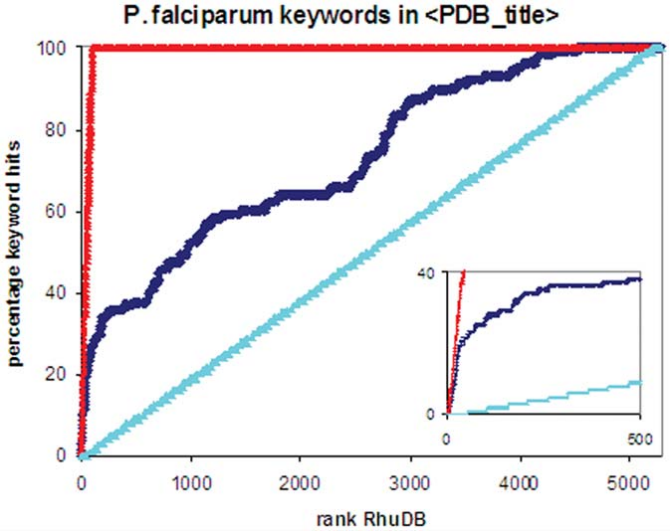
Enrichment of *P. falciparum* proteome by R_huDB_ – PDB targets. Enrichment curves were plotted as described in Figure 2. The 5283 protein malarial proteome was ranked by R_huDB_. *P. falciparum* and malaria related hits from PDB were identified using keyword searching of the 〈pdb_title〉 field. The enrichment percentage as a function of rank are shown in dark blue, while the red line shows an ideal case, and the light blue line indicates a random distribution. The insert highlights the first 500 entries.

**Figure 4:**
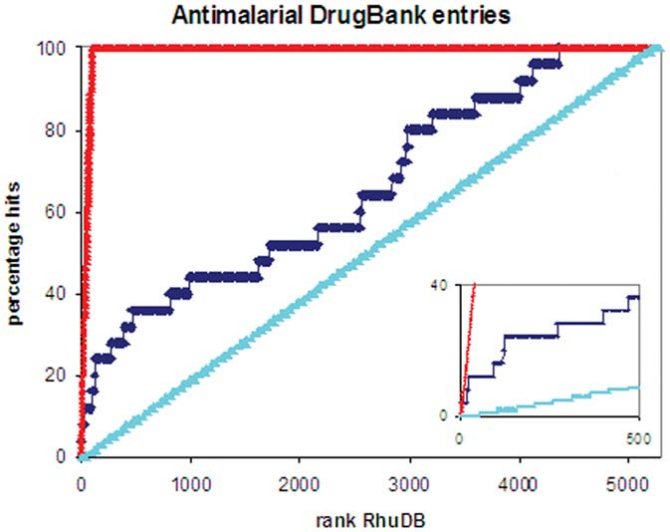
Enrichment of *P. falciparum* proteome by R_huDB_ – DrugBank targets. Enrichment curves were plotted as described in Figure 2. The 5283 protein malarial proteome was ranked by R_huDB_. *P. falciparum* and malaria related hits from DrugBank were identified using keyword searching of DrugBank website [4], as shown in supplementary Table S2 online. The 〈drugbank_title〉 field entries were matched to this list of *P. falciparum* or malaria related drug targets. The enrichment percentage as a function of rank are shown in dark blue, while the red line shows an ideal case, and the light blue line indicates a random distribution. The insert highlights the first 500 entries.

Similarly, query sequences homologous to known drug targets, as defined by DrugBank [4], but showing poor/no homology to any human protein, will have high R_huDB_ values. In Figure 3, the full *P. falciparum* proteome test set was ranked according to R_huDB_ and hits identified as having malaria related keywords in the best PDB match title, again indicating that high ranking entries are likely to be well characterised targets for drug discovery and development. Importantly, the same ranking showed good enrichment of known antimalarial drugs, as defined by DrugBank (Figure 4, see listed in supplementary Table S2 online). The DrugBank hits for each query sequence are listed at the bottom of the Microsoft Access form supplied in the output of Genomes2Drugs (see supplementary Figure S1B). These compounds include experimental small molecule drugs as well as FDA (Food and Drug Administration) approved medicinal drugs, which can be purchased and tested for *in vitro* effectivity [4]. After ranking the *P. falciparum* test set by R_huPDB_, 8 of the top 50 proteins showed homology to targets of FDA approved drugs. If an FDA approved drug is found to be effective against the pathogen of interest, a ‘change-of-application’ patent could be sought. As all the necessary toxicology, pharmacology and dosing analysis has already been completed, Phase IV clinical trials to confirm therapeutic use may be more rapidly instigated. This could become an extremely efficient and rapid route for drug development. With a lower financial barrier to entry, this strategy could be especially important in the development of therapeutic drugs against neglected infectious diseases affecting the developing world.

## Discussion

We have developed a free online resource that enriches any sized dataset of proteins of interest for those proteins likely to be most usefully in further drug discovery efforts. The program addresses the need to focus drug discovery effort on those protein targets that (i) do not show homology to proteins in the human genomes and (ii) show close homology to proteins for which the 3-dimentional structure is known. As an added feature, each input protein sequence is compared to the DrugBank set of known drug targets, and may identify known drugs that are able to inhibit the protein under investigation.

## Supporting Information

Figure S1 Screen shots of the input and output of the online Genomes2Drugs tool.
(0.58 MB PDF)

Table S1 Genomes2Drugs search of the Plasmodium falciparum proteome. The FASTA formatted proteome of the malarial parasite P. falciparum strain 3D7 was downloaded from EMBL-EBI Interg8. The Genomes2Drugs output was sorted by RhuPDB. Numerous fields have been removed and abridged for clarity. Putative, uncharacterised proteins likely to be good targets for further analysis are highlighted in blue. PDB homologue titles containing the word ‘plasmodium’ are highlighted in yellow. DrugBank hits associated with malaria, according to NCBI PubMed, are highlighted in green.
(0.05 MB PDF)

Table S2 DrugBank DrugCards with keywords “plasmodium” or “malaria”.
(0.01 MB PDF)
