# Supplementary material for: Genomes2Drugs: Identifies Target Proteins and Lead Drugs from Proteome Data
Source: PLoS One. 2009 Jul 10;4(7):e6195. doi: 10.1371/journal.pone.0006195 (PMC2704375; doi:10.1371/journal.pone.0006195)
Supplement: Figure S1 — Screen shots of the input and output of the online Genomes2Drugs tool. (0.58 MB PDF) [file pone.0006195.s001.pdf]

Supplementary data for Toomey *et al.* "Genomes2Drugs: identifies target proteins and lead drugs from proteome data".

[A]

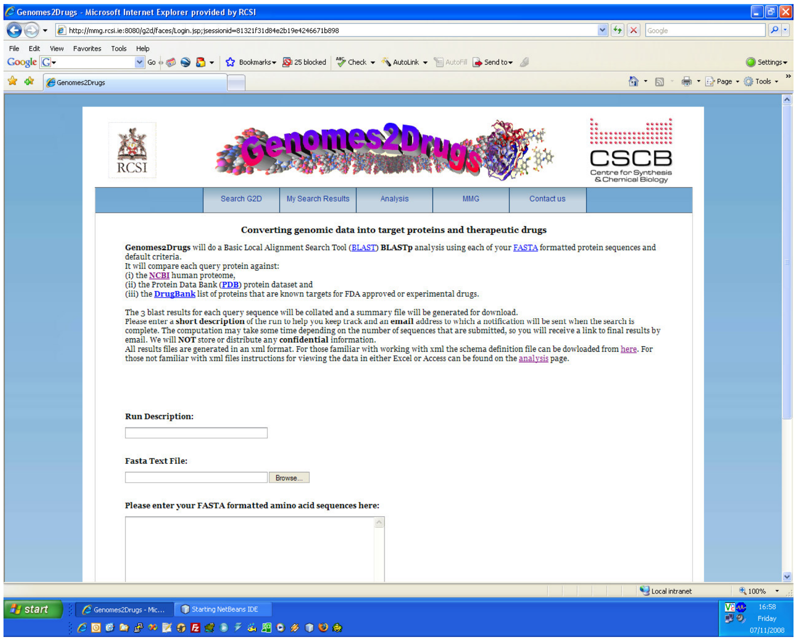

[B]

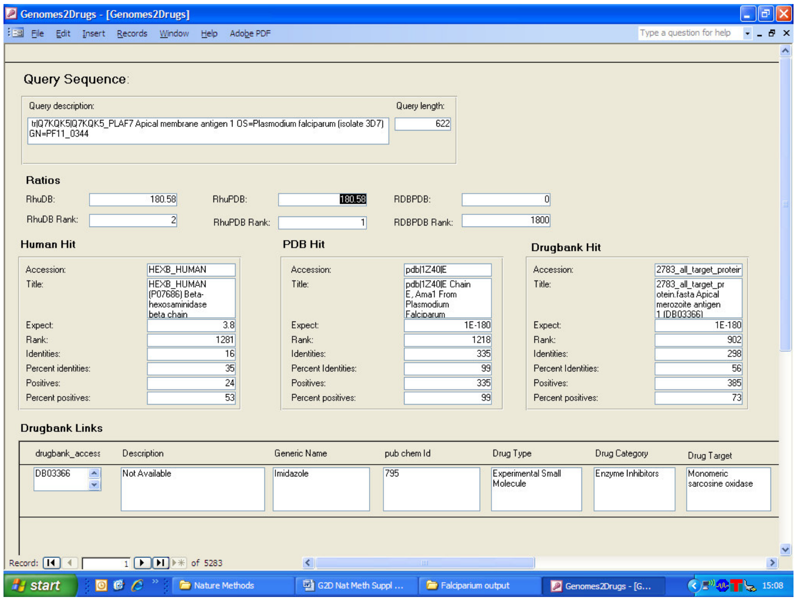

Supplementary Figure S1: *Screen shots of the input and output of the online Genomes2Drugs tool.*
