# Supplementary material for: Genomes2Drugs: Identifies Target Proteins and Lead Drugs from Proteome Data
Source: PLoS One. 2009 Jul 10;4(7):e6195. doi: 10.1371/journal.pone.0006195 (PMC2704375; doi:10.1371/journal.pone.0006195)
Supplement: Table S1 — Genomes2Drugs search of the Plasmodium falciparum proteome. The FASTA formatted proteome of the malarial parasite P. falciparum strain 3D7 was downloaded from EMBL-EBI Interg8. The Genomes2Drugs output was sorted by RhuPDB. Numerous fields have been removed and abridged for clarity. Putative, uncharacterised proteins likely to be good targets for further analysis are highlighted in blue. PDB homologue titles containing the word ‘plasmodium’ are highlighted in yellow. DrugBank hits associated with malaria, according to NCBI PubMed, are highlighted in green. (0.05 MB PDF) [file pone.0006195.s002.pdf]

Supplementary data for Toomey *et al.* "Genomes2Drugs: identifies target proteins and lead drugs from proteome data".

Supplementary Table S1: Genomes2Drugs search of the *Plasmodium falciparum* proteome.
The FASTA formatted proteome of the malarial parasite *P. falciparum* strain 3D7 was downloaded from EMBL-EBI Interg8. The Genomes2Drugs output was sorted by R_{huPDB}. Numerous fields have been removed and abridged for clarity. Putative, uncharacterised proteins likely to be good targets for further analysis are highlighted in blue. PDB homologue titles containing the word 'plasmodium' are highlighted in yellow. DrugBank hits associated with malaria, according to NCBI PubMed, are highlighted in green.

query_title	R_{huPDB}	R_{huPDB} Rank	human_accession	human_expect	pdb_title	pdb_expect	drugbank_title	drugbank_expect
tr|Q7KQK5|Q7KQK5_PLAF7 Apical membrane antigen 1	180.6	1	HEXB_HUMAN	3.8	pdb|1Z40|E Chain E, Ama1 From Plasmodium Falciparum	1E-180	2783_all_target_protein.fasta Apical merozoite antigen 1 (DB03366)	1E-180
tr|Q8IBE8|Q8IBE8_PLAF7 Erythrocyte binding antigen	178.4	2	Q8N9N0	0.026	pdb|1ZRO|B Chain B, Crystal Structure Of Eba-175 Region Ii (Rii) Crystallized In The Presence Of (Alpha)2,3-Sialyllactose	1E-180	1688_all_target_protein.fasta NADH dehydrogenase [ubiquinone] 1 subunit C2 (DB00157; DB01136)	0.48
tr|Q6LFB9|Q6LFB9_PLAF7 Enoyl-acyl carrier reductase	170.7	3	PECR_HUMAN	0.046	pdb|1VRW|B Chain B, Crystal Structure Analysis Of Plasmodium Falciparum Enoyl-Acyl-Carrier-Protein Reductase With Nadh	1E-172	44_all_target_protein.fasta Enoyl-acyl carrier reductase (DB01907)	1E-180
tr|Q8ILB1|Q8ILB1_PLAF7 NAD(P)H-dependent glutamate synthase, putative	165.9	4	DPYD_HUMAN	7E-15	pdb|1EA0|B Chain B, Alpha Subunit Of A. Brasilense Glutamate Synthase	1E-180	38_all_target_protein.fasta Ferredoxin-dependent glutamate synthase 2 (DB02926; DB03247; DB04184)	1E-180
tr|Q8IJH3|Q8IJH3_PLAF7 Orotidine 5'-phosphate decarboxylase	162.5	5	PYR5_HUMAN	0.32	pdb|2F84|A Chain A, Crystal Structure Of An Orotidine-5'-Monophosphate Decarboxylase Homolog From P.Falciparum	1E-163	2452_all_target_protein.fasta Orotidine 5'-phosphate decarboxylase (DB01915; DB02202; DB02309; DB02890; DB02957; DB03403; DB03685; DB03718)	0.0002
tr|Q8I5F4|Q8I5F4_PLAF7 Putative uncharacterized protein	156.4	6	AHNK_HUMAN	2.7	pdb|2B30|D Chain D, Initial Crystallographic Structural Analysis Of A Putative HadCOF-Like Hydrolase From Plasmodium Vivax	1E-156	4342_all_target_protein.fasta Phosphoglycolate phosphatase (DB01942)	2E-07
tr|Q8ILT0|Q8ILT0_PLAF7 NADP-specific glutamate dehydrogenase	151.5	7	DHE4_HUMAN	3E-29	pdb|2BMA|F Chain F, The Crystal Structure Of Plasmodium Falciparum Glutamate Dehydrogenase, A Putative Target For Novel Antimalarial Drugs	1E-180	67_all_target_protein.fasta Glutamate dehydrogenase (DB01907)	2E-36
tr|Q8ILJ7|Q8ILJ7_PLAF7 Phosphoenolpyruvate carboxylase	148.8	8	VTDB_HUMAN	6.3	pdb|1JQO|B Chain B, Crystal Structure Of C4-Form Phosphoenolpyruvate Carboxylase From Maize	1E-148	2554_all_target_protein.fasta Phosphoenolpyruvate carboxylase (DB04317)	1E-130
tr|Q8I3X4|Q8I3X4_PLAF7 Uridine phosphorylase, putative	143.5	9	UPP2_HUMAN	3	pdb|1Q1G|F Chain F, Crystal Structure Of Plasmodium Falciparum Pnp With 5'-Methylthio-Immucillin-H	1E-143	3815_all_target_protein.fasta Uridine phosphorylase, putative (DB03743; DB03881; DB04402)	1E-144
tr|Q76NM3|Q76NM3_PLAF7 L-lactate dehydrogenase	141.8	10	LDHC_HUMAN	6E-39	pdb|1CEQ|A Chain A, Chloroquine Binds In The Cofactor Binding Site Of Plasmodium Falciparum Lactate Dehydrogenase	1E-180	2579_all_target_protein.fasta L-lactate dehydrogenase (DB01907; DB02111; DB02401; DB02737; DB03162; DB03940; DB04640; DB04641)	1E-180

tr|Q8IB03|Q8IB03_PLAF7 Putative uncharacterized protein PF08_0063	138.6	11	CLPB_HUMAN	4E-42	pdb|1QVR|C Chain C, Crystal Structure Analysis Of Clpb	1E-180	1453_all_target_protein.fasta Chaperone clpB (DB04395)	1E-180
tr|Q6LFF9|Q6LFF9_PLAF7 Transketolase, putative	138.3	12	TKT_HUMAN	2E-42	pdb|1GPU|B Chain B, Transketolase Complex With Reaction Intermediate	1E-180	1877_all_target_protein.fasta Transketolase 1 (DB01658; DB01682; DB01987; DB02814; DB03937)	1E-180
tr|Q8IM16|Q8IM16_PLAF7 Plasmepsin, putative	135.9	13	CATD_HUMAN	8E-45	pdb|1LS5|B Chain B, Crystal Structure Of Plasmepsin Iv From P. Falciparum In Complex With Pepstatin A	1E-180	2060_all_target_protein.fasta Plasmepsin-2 (DB02505; DB03063; DB04373; DB04378)	1E-161
tr|Q8IDR1|Q8IDR1_PLAF7 Phosphoenolpyruvate	135.4	14	PTN13_HUMAN	2.7	pdb|1II2|B Chain B, Crystal Structure Of Phosphoenolpyruvate Carboxykinase (Pepck) From Trypanosoma Cruzi	1E-135	1716_all_target_protein.fasta Phosphoenolpyruvate carboxykinase [ATP] (DB02737; DB03431; DB04184)	1E-113
tr|Q8I6V3|Q8I6V3_PLAF7 Plasmepsin 2	132.3	15	CATD_HUMAN	2E-48	pdb|2BJU|A Chain A, Plasmepsin Ii Complexed With A Highly Active Achiral Inhibitor	1E-180	2060_all_target_protein.fasta Plasmepsin-2 (DB02505; DB03063; DB04373; DB04378)	1E-180
sp|Q7KQM4|PLM1_PLAF7 Plasmepsin-1	129.5	16	RENI_HUMAN	3E-51	pdb|2BJU|A Chain A, Plasmepsin Ii Complexed With A Highly Active Achiral Inhibitor	1E-180	2060_all_target_protein.fasta Plasmepsin-2 (DB02505; DB03063; DB04373; DB04378)	1E-180
tr|Q8ILF7|Q8ILF7_PLAF7 Glutamate dehydrogenase, putative	126.0	17	DHE4_HUMAN	1E-38	pdb|2BMA|F Chain F, The Crystal Structure Of Plasmodium Falciparum Glutamate Dehydrogenase, A Putative Target For Novel Antimalarial Drugs	1E-164	67_all_target_protein.fasta Glutamate dehydrogenase (DB01907)	6E-51
tr|Q8IM15|Q8IM15_PLAF7 HAP protein GN=PF14_0078	119.6	18	RENI_HUMAN	4E-36	pdb|2BJU|A Chain A, Plasmepsin Ii Complexed With A Highly Active Achiral Inhibitor	1E-155	2060_all_target_protein.fasta Plasmepsin-2 (DB02505; DB03063; DB04373; DB04378)	1E-155
tr|Q8IJA9|Q8IJA9_PLAF7 Adenosine deaminase, putative	116.3	19	ADA_HUMAN	2E-22	pdb|2AMX|B Chain B, Crystal Structure Of Plasmodium Yoelii Adenosine Deaminase (Py02076)	1E-138	4308_all_target_protein.fasta Adenosine deaminase (DB02096; DB02616; DB02830; DB03015; DB03220; DB03370; DB03572)	8E-26
tr|Q8IIJ8|Q8IIJ8_PLAF7 Heat shock protein 101, putative	114.0	20	CLPB_HUMAN	1E-37	pdb|1QVR|C Chain C, Crystal Structure Analysis Of Clpb	1E-151	1453_all_target_protein.fasta Chaperone clpB (DB04395)	1E-152
tr|Q8I2Z8|Q8I2Z8_PLAF7 6-phosphofructokinase, putative	111.3	21	K6PL_HUMAN	0.002	pdb|1KZH|B Chain B, Structure Of A Pyrophosphate-Dependent Phosphofructokinase From The Lyme Disease Spirochete Borrelia Burgdorferi	1E-114	1300_all_target_protein.fasta 6-phosphofructokinase (DB02726; DB04493)	8E-11
sp|Q08210|PYRD_PLAF7 Dihydroorotate dehydrogenase homolog, mitochondrial	110.3	22	PYRD_HUMAN	2E-70	pdb|1TV5|A Chain A, Plasmodium Falciparum Dihydroorotate Dehydrogenase With A Bound Inhibitor	1E-180	3153_all_target_protein.fasta Dihydroorotate dehydrogenase homolog, mitochondrial (DB00263; DB00359; DB00664; DB01145)	1E-180
tr|Q8I2S7|Q8I2S7_PLAF7 3-oxoacyl-(Acyl-carrier protein) reductase, putative	106.6	23	DHB8_HUMAN	4E-43	pdb|2C07|A Chain A, Oxoacyl-Acp Reductase Of Plasmodium Falciparum	1E-149	3052_all_target_protein.fasta 3-oxoacyl-[acyl-carrier-protein] reductase (DB03461)	7E-66
tr|Q8IEU2|Q8IEU2_PLAF7 Plasmodium falciparum gamete antigen 27/25	105.7	24	Q5R336	4.6	pdb|1N81|A Chain A, Crystal Structure Of Pfg27 From Plasmodium Falciparum	1E-105	2135_all_target_protein.fasta Voltage-dependent L-type calcium channel subunit alpha-1C (DB00270; DB00308; DB00622; DB00653; DB00661; DB01388)	1.1

tr|Q8I5P5|Q8I5P5_PLAF7 Glycerol-3-phosphate dehydrogenase, putative	103.0	25	Q8N335	1E-77	pdb|1YJ8|C Chain C, Initial Structural Analysis Of Plasmodium Falciparum Glycerol-3-Phosphate Dehydrogenase	1E-180	4420_all_target_protein.fasta Glycerol-3-phosphate dehydrogenase [NAD+], cytoplasmic (DB00157)	5E-75
tr|Q8I377|Q8I377_PLAF7 ATP-dependent heat shock protein, putative	102.3	26	CLPX_HUMAN	2E-09	pdb|1DO0|F Chain F, Orthorhombic Crystal Form Of Heat Shock Locus U (Hslu) From Escherichia Coli	1E-111	2254_all_target_protein.fasta ATP-dependent hsl protease ATP-binding subunit hslU (DB04395)	1E-111
tr|Q8I4Y0|Q8I4Y0_PLAF7 Kinesin-like protein, putative	99.8	27	Q5T7B7	6E-81	pdb|1RY6|A Chain A, Crystal Structure Of Internal Kinesin Motor Domain	1E-180	1827_all_target_protein.fasta Kinesin-like protein KIF2C (DB03431; DB04395)	3E-79
tr|Q8I3W2|Q8I3W2_PLAF7 Ribose 5-phosphate epimerase, putative	97.3	28	RPIA_HUMAN	2E-28	pdb|2F8M|B Chain B, Ribose 5-Phosphate Isomerase From Plasmodium Falciparum	1E-125	180_all_target_protein.fasta Ribose-5-phosphate isomerase A (DB01756)	1E-29
tr|Q76NM5|Q76NM5_PLAF7 Erythrocyte binding antigen 140	97.3	29	REST_HUMAN	9	pdb|1ZRO|B Chain B, Crystal Structure Of Eba-175 Region Ii (Rii) Crystallized In The Presence Of (Alpha)2,3-Sialyllactose	5E-97	359_all_target_protein.fasta NADH dehydrogenase [ubiquinone] 1 alpha subcomplex subunit 10, mitochondrial (DB00157)	3.2
tr|Q6LEZ4|Q6LEZ4_PLAF7 Putative 6-pyruvoyl tetrahydropterin synthase (6-pyruvoyl tetrahydropterin synthase, putative)	96.1	30	BRD7_HUMAN	0.4	pdb|1Y13|C Chain C, Structural Analysis Of Plasmodium Falciparum 6-Pyruvoyl Tetrahydropterin Synthase (Ptps)	3E-97	52_all_target_protein.fasta 6-pyruvoyl tetrahydrobiopterin synthase (DB03886)	0.13
sp|Q8ILQ7|GST_PLAF7 Glutathione S-transferase	93.9	31	PTGD2_HUMAN	7E-18	pdb|2AAW|C Chain C, Studies On Ligand Binding And Enzyme Inhibition Of Plasmodium Falciparum Glutathione S-Transferase	1E-111	2834_all_target_protein.fasta Glutathione S-transferase (DB01942; DB04132)	1E-112
tr|Q8IJI7|Q8IJI7_PLAF7 Deoxyribose-phosphate aldolase, putative	92.6	32	DEOC_HUMAN	4E-22	pdb|2A4A|B Chain B, Deoxyribose-Phosphate Aldolase From P. Yoelii	1E-114	4149_all_target_protein.fasta Deoxyribose-phosphate aldolase (DB04087)	4E-32
tr|Q8ILQ2|Q8ILQ2_PLAF7 Glutathione reductase	91.3	33	GSHR_HUMAN	2E-89	pdb|1ONF|A Chain A, Crystal Structure Of Plasmodium Falciparum Glutathione Reductase	1E-180	1508_all_target_protein.fasta Glutathione reductase (DB03147)	1E-180
sp|Q7KQM0|TPIS_PLAF7 Triosephosphate isomerase	87.3	34	Q6FHP9	2E-58	pdb|1O5X|B Chain B, Plasmodium Falciparum Tim Complexed To 2-Phosphoglycerate	1E-145	3437_all_target_protein.fasta Triosephosphate isomerase (DB01709; DB01779; DB02515; DB02726; DB02951; DB04510)	1E-146
tr|Q8I372|Q8I372_PLAF7 Formylmethionine deformylase, putative	85.6	35	DEFM_HUMAN	4E-16	pdb|1RL4|B Chain B, Plasmodium Falciparum Peptide Deformylase Complex With Inhibitor	1E-101	2132_all_target_protein.fasta Formylmethionine deformylase, putative (DB02625; DB03648)	1E-139
tr|Q8I6T4|Q8I6T4_PLAF7 Beta-hydroxyacyl-acp dehydratase	84.4	36	TNFL6_HUMAN	4.8	pdb|1Z6B|F Chain F, Crystal Structure Of Plasmodium Falciparum Fabz At 2.1 A	2E-84	1863_all_target_protein.fasta 3-hydroxydecanoyl-[acyl-carrier-protein] dehydratase (DB03813)	0.12
tr|Q3V7I1|Q3V7I1_PLAF7 Putative pyridoxine biosynthetic enzyme pdx1 homologue (Pyridoxine biosynthetic enzyme pdx1 homologue, putative)	83.4	37	GOGB1_HUMAN	0.24	pdb|1ZNN|F Chain F, Structure Of The Synthase Subunit Of Plp Synthase	1E-84	1988_all_target_protein.fasta Modification methylase RsrI (DB01752; DB01910; DB02282)	0.92
tr|O77330|O77330_PLAF7 Asparagine synthetase, putative	82.7	38	ASNS_HUMAN	5E-80	pdb|1CT9|D Chain D, Crystal Structure Of Asparagine Synthetase B From Escherichia Coli	1E-162	2840_all_target_protein.fasta Asparagine synthetase [glutamine-hydrolyzing] (DB00128; DB00130; DB00142; DB00171; DB00174)	7E-81
tr|Q8I5L3|Q8I5L3_PLAF7 D-ribulose-5-phosphate 3-epimerase, putative	82.5	39	RPE_HUMAN	3E-52	pdb|1TQX|B Chain B, Crystal Structure Of Pfal009167 A Putative D-Ribulose 5- Phosphate 3-Epimerase From P.Falciparum	1E-134	1605_all_target_protein.fasta Delta 1-pyrroline-5-carboxylate synthetase (DB00142)	0.43

tr|Q8I1R6|Q8I1R6_PLAF7 Bifunctional dihydrofolate reductase-thymidylate synthase	81.9	40	TYSY_HUMAN	8E-99	pdb|1J3K|D Chain D, Quadruple Mutant (N51i+c59r+s108n+i164l) Plasmodium Falciparum Dihydrofolate Reductase-Thymidylate Synthase (Pfdhfr-Ts) Complexed With Wr99210, Nadph, And Dump	1E-180	1991_all_target_protein.fasta Bifunctional dihydrofolate reductase-thymidylate synthase (DB00205; DB01131)	1E-180
tr|Q8IJS1|Q8IJS1_PLAF7 Hypoxanthine phosphoribosyltransferase	81.0	41	HPRT_HUMAN	9E-54	pdb|1CJB|D Chain D, Malarial Purine Phosphoribosyltransferase	1E-134	4516_all_target_protein.fasta Hypoxanthine-guanine-xanthine phosphoribosyltransferase (DB02075; DB02212)	1E-134
tr|Q8IBG7|Q8IBG7_PLAF7 Antioxidant protein, putative	80.0	42	PRDX5_HUMAN	2E-14	pdb|1XIY|B Chain B, Crystal Structure Of Plasmodium Falciparum Antioxidant Protein (1-Cys Peroxiredoxin)	2E-94	1225_all_target_protein.fasta Peroxiredoxin (DB03661)	6E-97
tr|Q8IDF6|Q8IDF6_PLAF7 Adenylosuccinate synthetase	80.0	43	PURA2_HUMAN	1E-100	pdb|1P9B|A Chain A, Structure Of Fully Ligated Adenylosuccinate Synthetase From Plasmodium Falciparum	1E-180	1014_all_target_protein.fasta Adenylosuccinate synthetase (DB02109; DB03510; DB04315)	1E-180
sp|Q7KQL9|ALF_PLAF7 Fructose-bisphosphate aldolase	78.0	44	ALDOC_HUMAN	1E-102	pdb|1A5C|B Chain B, Fructose-1,6-Bisphosphate Aldolase From Plasmodium Falciparum	1E-180	1012_all_target_protein.fasta Fructose-bisphosphate aldolase A (DB02512; DB04326; DB04733)	1E-97
tr|Q8II92|Q8II92_PLAF7 Deoxyuridine 5'-triphosphate nucleotidohydrolase, putative	76.4	45	DUT_HUMAN	7E-08	pdb|1VYQ|C Chain C, Novel Inhibitors Of Plasmodium Falciparum Dutpase Provide A Platform For Anti-Malarial Drug Design	3E-84	2909_all_target_protein.fasta Deoxyuridine 5'-triphosphate nucleotidohydrolase, putative (DB04685)	2E-84
tr|Q8I2M1|Q8I2M1_PLAF7 Guanylate kinase, putative	75.3	46	Q5T435	2E-32	pdb|1Z6G|A Chain A, Crystal Structure Of Guanylate Kinase From Plasmodium Falciparum	1E-107	460_all_target_protein.fasta Guanylate kinase (DB01972; DB03431)	1E-35
tr|Q8IE02|Q8IE02_PLAF7 Apurinic/apyrimidinic endonuclease Apn1	74.9	47	RARB_HUMAN	1.5	pdb|1QUM|A Chain A, Crystal Structure Of Escherichia Coli Endonuclease Iv In Complex With Damaged Dna	2E-75	137_all_target_protein.fasta Retinoic acid receptor beta (DB02877)	0.18
tr|Q8IKG4|Q8IKG4_PLAF7 1-deoxy-D-xylulose 5-phosphate reductoisomerase	73.3	48	No hit found	1000	pdb|1R0L|D Chain D, 1-Deoxy-D-Xylulose 5-Phosphate Reductoisomerase From Zymomonas Mobilis In Complex With Nadph	5E-71	2730_all_target_protein.fasta 1-deoxy-D-xylulose 5-phosphate reductoisomerase (DB04184)	9E-72
tr|Q8IDI8|Q8IDI8_PLAF7 Putative uncharacterized protein MAL13P1.257	72.5	49	Q9NWV4	3E-14	pdb|1ZSO|B Chain B, Hypothetical Protein From Plasmodium Falciparum	1E-86	349_all_target_protein.fasta Isovaleryl-CoA dehydrogenase, mitochondrial (DB03147; DB04036)	0.25
sp|Q8IAY6|SODF_PLAF7 Superoxide dismutase [Fe]	70.6	50	Q5TCM1	4E-38	pdb|2AWP|B Chain B, Crystal Structure Of Plasmodium Knowlesi Structure Of Iron Super-Oxide Dismutase	1E-108	568_all_target_protein.fasta Superoxide dismutase [Mn], mitochondrial (DB04436)	7E-39
