# Supplementary material for: Genomes2Drugs: Identifies Target Proteins and Lead Drugs from Proteome Data
Source: PLoS One. 2009 Jul 10;4(7):e6195. doi: 10.1371/journal.pone.0006195 (PMC2704375; doi:10.1371/journal.pone.0006195)
Supplement: Table S2 — DrugBank DrugCards with keywords “plasmodium” or “malaria”. (0.01 MB PDF) [file pone.0006195.s003.pdf]

Supplementary data for Toomey *et al.* "Genomes2Drugs: identifies target proteins and lead drugs from proteome data".

Supplementary Table S2: DrugBank DrugCards with keywords "plasmodium" or "malaria".

**DB number:**	**Name:**
DB00205	Pyrimethamine
DB00250	Dapsone
DB00254	Doxycycline
DB00256	Lymecycline
DB00263	Sulfisoxazole
DB00358	Mefloquine
DB00453	Clomocycline
DB00468	Quinine
DB00608	Chloroquine
DB00613	Amodiaquine
DB00618	Demeclocycline
DB00664	Sulfametopyrazine
DB01087	Primaquine
DB01117	Atovaquone
DB01131	Proguanil
DB01218	Halofantrine
DB01299	Sulfadoxine
DB01611	Hydroxychloroquine
